# Development of NMDA receptors contributes to the enhancement of electroencephalogram oscillations under volatile anesthetics in rats

**DOI:** 10.3389/fncir.2022.1065374

**Published:** 2022-12-15

**Authors:** Mingyue Zhang, Yali Chen, Jin Liu, Yaoxin Yang, Rurong Wang, Donghang Zhang, Tao Zhu

**Affiliations:** ^1^Department of Anesthesiology, West China Hospital of Sichuan University, Chengdu, China; ^2^Laboratory of Anesthesia and Critical Care Medicine, National-Local Joint Engineering Research Centre of Translational Medicine of Anesthesiology, West China Hospital of Sichuan University, Chengdu, China

**Keywords:** electroencephalogram, NMDA development, slow-delta, theta, alpha, volatile anesthetics

## Abstract

**Background:**

Volatile anesthetics including sevoflurane and isoflurane enhance oscillations of cortical electroencephalogram (EEG), partly by their modulations on glutamate-mediated excitatory synaptic transmission. Expression of NMDA receptors is increased during neonatal development. However, how the development of NMDA receptors influences EEG under volatile anesthesia remains unclear.

**Methods:**

Expressions of NMDA receptor subtypes (NR1, NR2A, and NR2B) during neonatal development were measured by Western blotting. MAC (minimal alveolar concentration) of isoflurane and sevoflurane that inducing loss of righting reflex (LORR) and no response to tail-clamp (immobility) were measured to verify the effect of NR1 expression on anesthetic potency during neonatal development. Cortical electroencephalogram recording was used to examine the influence of NR1 expression on the power density of EEG.

**Results:**

The expressions of GluNR1, GluNR2A and GluNR2B receptors were gradually increased during neonatal development in cortex, hippocampus and thalamus of rats. Knockdown of NR1 enhanced the sedative potency of volatile anesthetics but not on immobility potency in postnatal day 14 (P14)-P17 rats. For cortical EEG, along with the increased concentration of volatile anesthetics, cortical slow-delta oscillations of P5 rats were inhibited, theta and alpha oscillations were not changed significantly; while these oscillations were enhanced until high anesthetic concentrations in P21 rats. Knockdown of NR1 in forebrain suppressed the enhancement of cortical EEG oscillations in P21 rats.

**Conclusion:**

The development of NMDA receptors may contribute to the enhancement of cortical EEG oscillations under volatile anesthetics.

## Introduction

The mechanisms by which volatile anesthetics function remain unclear. Currently, more than 300 million people receive general anesthesia each year; therefore, the importance of anesthetic safety is significant and at least partly depends on an understanding of anesthetic mechanisms (Meara et al., 2016). There is a consensus that the volatile anesthetics may disrupt the balance between excitatory and inhibitory neurotransmissions (Barker and Ransom, 1978; Jones et al., 1992).

Owing to its non-invasive nature, electroencephalogram (EEG) has become a basic clinical tool for monitoring anesthetic status in patients (Forgacs et al., 2014). EEG measures the electrical oscillations of brain activity and offers a general reflection of the electrophysiological activity of the brain in the cerebral cortex or scalp surface (Malver et al., 2014). There are different component oscillations in the EEG at different ages. Under sevoflurane, slow-delta oscillations are present in the EEG at all ages after birth, but theta and alpha oscillations appear at 4 months of life (Cornelissen et al., 2015). The divergence in EEG oscillations reflected by different ages may be related to differences in regional synapse formation in the central nervous system and changes in metabolism (Brody et al., 1987). Interestingly, different general anesthetics induce varied EEG signatures, which reflect their distinct modulatory effects on the central nervous system (Purdon et al., 2015). For example, loss of consciousness caused by propofol is accompanied by obvious slow-delta and alpha oscillations in the EEG (Gugino et al., 2001). Ketamine, a non-competitive antagonist of the N-methyl-D-aspartate (NMDA) receptor, induces an increase in EEG activity of theta oscillations (Saletu et al., 1975). The EEG signatures of dexmedetomidine include slow-delta oscillations and spindles (Purdon et al., 2015). Under Volatile anesthetics, which suggested to act on multiple targets, such as GABAA receptors, NMDA receptors, and two-pore-domain potassium channels (Hemmings et al., 2005), alpha, slow-delta, and theta oscillations appear along with the unconsciousness (Purdon et al., 2015).

NMDA receptors have diverse but overlapping regional expression modes (Cull-Candy and Leszkiewicz, 2004). This suggests a specific role for NMDA subtypes in different brain regions during development. The structure of the NMDA receptor is composed of four subunits derived from three major subtypes: GluNR1, GluNR2A-B, and GluNR3A-B (Yamakura and Shimoji, 1999). It remains unclear whether the differences in the development of these subunits affect brain development. Previous studies have shown that NR1 expression starts as early as E14, reaches a peak at approximately the third week of life, and then decreases lightly to adult levels in splicing of the rat brain (Laurie and Seeburg, 1994). Individual subtypes also broadly follow this pattern (Paupard et al., 1997). As an obligatory NMDA receptor channel-forming subunit, GluNR1 is widely expressed in the central nervous system. Mutations in the GluNR1 subunit reduce the sensitivity to volatile anesthetics (Ogata et al., 2006). Interestingly, the effect of anesthetics on NMDA is rarely dependent on GluRN3 (Yamakura et al., 2005). Therefore, among the subunits, GluNR1 seems to be appropriate candidates to test the relation between development of NMDA receptors and EEG oscillations under volatile anesthetics in rats.

In the present study, we aimed to investigate the hypothesis that the enhancement of EEG power under volatile anesthetics may require mature NMDA-mediated glutamatergic neurotransmission. Meanwhile, understanding the EEG characteristics under volatile anesthesia during development as well as the underlying mechanism will improve the monitor of anesthesia depth and promote the safety of pediatric anesthesia in clinical practice.

## Materials and methods

### Animals

All animal care procedures in this study were approved by the Animal Ethics Committee of West China Hospital of Sichuan University (#2018191A, Dec. 28th, 2018; Chengdu, China), and all protocols adhered to the Animal Research Reporting *In vivo* Experiments (ARRIVE) guidelines. Sprague-Dawley rats (P5: *n* = 25, 12/13 male/female; P7: *n* = 12, 6/6 male/female; P11: *n* = 10, 6/4 male/female; P21: *n* = 25, 12/13 male/female) were used. The sex of the rats was confirmed by observing the anogenital distance (LaPrairie and Murphy, 2007). All animals were purchased from Chengdu Dassy Biological Technology Co. Ltd. (Chengdu, China) and kept in cages with their littermates and mothers under standard conditions (22-24°C, humidity 45-55%, lights on from 7:00 to 19:00). All efforts were made to minimize animal suffering and reduce the number of animals used.

### Western blotting for NR1, NR2A, and NR2B

The rats were deeply anesthetized with 2% isoflurane and perfused with phosphate-buffered saline through the left cardiac ventricle. Their cortices, hippocampi, and thalami were removed from their skulls, placed in liquid nitrogen, and stored at −80°C until further use. Total protein from the cortex, hippocampus, and thalamus was isolated using RIPA buffer (Tris-HCl 50 mM, NaCl 150 mM, Sodium deoxycholate 0.5%, and Triton 1%) with protease inhibitors. Protein concentrations of the samples were quantified using a BCA protein assay kit (Beyotime, Shanghai, China). The prepared protein samples were separated by 12% SDS-PAGE and transferred to a polyvinylidene fluoride membrane (Bio-Rad, Hercules, CA, United States). After blocking with 5% skim milk for 2 h, membranes were washed three times (10 min/wash) and incubated with primary antibodies overnight at 4°C, including anti-NR1 (1:1,000 dilution, GeneTex, Texas, US), anti-NR2A (1:1,000 dilution, Abcam, Cambridge, UK), anti-NR2B (1:1,000 dilution, MilliporeSigma, Burlington, MA, United States), and anti-β-actin (1:5,000, Abcam, Cambridge, UK). The next day, the membrane was washed three times and incubated with the secondary antibody (1:4,000, Cell Signaling Technology, Beverly, MA, United States) for 2 h. Finally, the membrane was removed using a gel imager (Amersham Imager 600, United States). The intensities of the bands were analyzed using ImageJ software (National Institutes of Health, Bethesda, MD, United States).

### siRNA injection

To verify NR1 knockdown efficiency, NR1-siRNA was bilaterally injected into the lateral cerebroventricle of P3 rats, and samples were collected at P5 for the Western blotting (WB) experiment. To investigate the effect of NR1 on sensitivity to volatile anesthetics, NR1-siRNA was bilaterally injected into the lateral cerebroventricle of P7 rats, and siRNA was injected again on the 14th day of birth to maintain the knockdown effect of NR1. NR1-siRNA (5′-GCACACUGGACUCAUUUAUTT-3′) and control-siRNA (5′ -UUCUCCGAACGUGUCACGUTT -3′) were dissolved in RNase-free water and injected as a 1:1 mixture with *in vivo* SilenceMagTM transfection reagent (OZ Biosciences, Marseille, France). Rats less than 7 days old were anesthetized by cold immersion (Chakrabarty et al., 2013), and older rats were anesthetized by inhalation anesthesia with 2% isoflurane. After anesthesia, the rats were fixed in a stereoscopic locator, and the scalp was cut open to expose the bregma. For P3 rats, NR1-siRNA or control-siRNA (1 μl) was injected vertically at the coordinates: AP = −1 mm, ML = ± 1.5 mm, DV = −3.5 mm. For P7 rats, NR1-siRNA or control-siRNA (2 μl) was injected at the following coordinates: AP = −1 mm, ML = ± 1.6 mm, DV = −3.6 mm. For P14/P17 rats, NR1-siRNA or control-siRNA (2 μl) was injected at the following coordinates: AP = −1 mm, ML = ± 2.5 mm, DV = −5.0 mm. The injection speed was 500 nl min-1. To allow siRNA spreading and avoid spillage, the injector was left in the injection site for at least 5 min (Clayton et al., 2021).

### Behavioral assays

To determine the sedative effect of inhalation anesthetics on rats, we compared the loss of righting reflex (LORR) between the control and NR1-siRNA groups. If the rats did not turn over within 60 s after anesthesia, we defined it as LORR (Chen et al., 2021). Righting reflex latency was measured by recording the time required for the rat to roll over (Koyama et al., 2013). To determine the immobility of volatile anesthetics in rats, the withdrawal response to tail clamping was used. An alligator clip (type 85, Newark Electronics, United States) was placed at the base of the animal’s tail. If the rats did not move within 60 s, we referred to this as immobility (Rau et al., 2011). The latency of the withdrawal response to tail clamping was measured by recording the time required for the rats to move (Koyama et al., 2016). We used three isolated cylindrical chambers for the experiments (15 cm in length and 5 cm in diameter), and each cylinder was placed with control group rats and NR1-siRNA group rats. Throughout the experiment, the rectal temperatures of the rats were maintained between 36°C and 38°C using a heating blanket. The chamber was continuously flushed with 100% oxygen (2.0 L/min). The outlet concentrations of isoflurane (RWD Life Science, Shenzhen, China), sevoflurane/isoflurane (Abbott Pharmaceutical Co., Ltd., China), O2, and CO2 were continuously monitored using a gas analyzer (Philips HP M1026B). In the LORR experiment, rats were first exposed to inhalation anesthesia at an initial concentration of 0.3% isoflurane or 0.6% sevoflurane for 30 min. The cylinder was then rotated by 180°. If the rats were able to turn themselves prone onto all four limbs, we increased the concentration of inhalation anesthesia by 0.05% for another 20-min equilibration period, and the response was tested again until the rats failed to turn over. The MAC values were recorded at this time. In the immobility experiment, isoflurane (1.6%) or sevoflurane (2.0%) was administered for 30 min. If movement was observed, the concentrations of isoflurane and sevoflurane were increased as the LORR experiment described, until no somatotropic reaction occurred. To test the latency of the righting reflex and withdrawal response to tail clamping, we used a concentration of isoflurane and/or sevoflurane that was ∼0.2% lower than the average MAC (mean MAC between the control and NR1-siRNA groups). After 30 min of anesthesia exposure, the time of turning over or that of body movement was recorded. If there was no response within 60 s, then we recorded the upper limit of 60 s (Koyama et al., 2013).

### EEG recording

Three hand-made EEG electrodes were implanted in each animal. Using bregma as a reference, the coordinates of two recording electrodes in the frontal cortex were AP = + 1.5 mm, ML = ± 1.5 mm, and the coordinates of the grounding electrode were AP = −1.5 mm, ML = −1.5 mm. The electrodes were attached to the dura mater. At the start of the experiment, each rat was placed in a cuboid container (25 cm in length,15 cm in width, and 12 cm in height) with a heating blanket under the chamber to keep the body warm. The chamber was continuously flushed with O2 (100%) at a flow rate of 2 L min^–1^. To record the changes in EEG with increasing concentrations of inhalation anesthesic at different ages, we used the concentrations of sevoflurane and isoflurane measured in previous behavioral experiments: 50% MAC_LORR_, MAC_LORR_, and MAC_*immobility*_. In the experiment comparing EEG changes between the NR1-siRNA and control groups, we used the MAC_LORR_ of sevoflurane and isoflurane. Each isoflurane concentration was maintained for at least 20 min. The outlet concentrations of isoflurane (Abbott Pharmaceutical Co., Ltd., China) and/or sevoflurane (Abbott Pharmaceutical Co., Ltd., China) were continuously monitored using a gas monitor (M1026B, Philips HP, United States).

### Processing of cortex EEG

Cortical EEG signals were recorded using a Pinnacle EEG recording system (Part#8200-SL; Pinnacle Technology, United States). EEG was recorded for at least 10 min at rest, followed by inhalation anesthesia for 20 min, combined with LORR behavior. EEG data were manually validated and accepted if the signal was of good quality in the resting state and under inhalation anesthesia (P5:20 s; P11 and P21:60 s). Because absolute power is sensitive to changes in the amount of total energy contained in the EEG signal, we mainly analyzed the power density of slow-delta (0-4 Hz), theta (5-8 Hz), and alpha (9-12 Hz). The EEG signals were analyzed offline as previously described (Gui et al., 2021). The parameters for coherence analysis were set as follows: window length, T = 4 s with 0-s overlap; time-oscillation width product, TW = 3; number of tapers, K = 5; and a spectral resolution of 2 W of 1.5 Hz (Jiang et al., 2022). Raw signals were pre-amplified, digitized, and recorded using a Sirenia Acquisition system (Part #8206-SL, Pinnacle Technology, United States) and analyzed using MATLAB (version 2006a, MathWorks, United States).

### Statistical analysis

Values are reported as the mean ± standard deviation (SD). GraphPad Prism version 8.0 software (GraphPad Software, CA, United States) was used for the statistical analysis. Sample size analysis was performed using PASS 15 software (NCSS, LLC, Kaysville, UT, United States). By the preliminary test (*n* = 4) on the difference of MAC_LORR_ between the NR1-siRNA group and the control group on P14 rats (isoflurane:0.56% ± 0.04% vs. 0.70% ± 0.1%; sevoflurane:1.06% ± 0.05% vs.1.25% ± 0.10%), the calculated minimal sample size was four for each group (α = 0.05, β = 0.10). In EEG experiments for the difference on power density of theta oscillations under volatile anesthesia between the NR1-siRNA group and the control group on P21 rats (isoflurane:34.44 ± 9.75 μV^2^ Hz^–1^ vs. 44.22 ± 17.08 μV^2^ Hz^–1^; sevoflurane:29.09 ± 6.49μV^2^ Hz^–1^vs. 43.33 ± 11.63μV^2^ Hz^–1^), the minimal sample size was four (α = 0.05, β = 0.10), and a sample size of five was chosen. In terms of statistical method selection, WB experiments were performed using ordinary one-way analysis of variance (ANOVA) followed by Tukey’s multiple comparison test. Two-way ANOVA was used for statistical analysis of the power density of EEG at different ages under physiological conditions and behavior experiments. Changes in the EEG power density at the same age group were compared using paired *t*-tests. The change ratio of EEG oscillations between two groups was performed using unpaired two-tailed t-tests The exact statistical methods employed are indicated in the figure legends, and a P value less than 0.05 was considered significant.

## Results

The levels of NR1, NR2A, and NR2B in the cortex, hippocampus, and thalamus increased gradually after birth, and the power density of the EEG was progressively enhanced.

**FIGURE 1:**
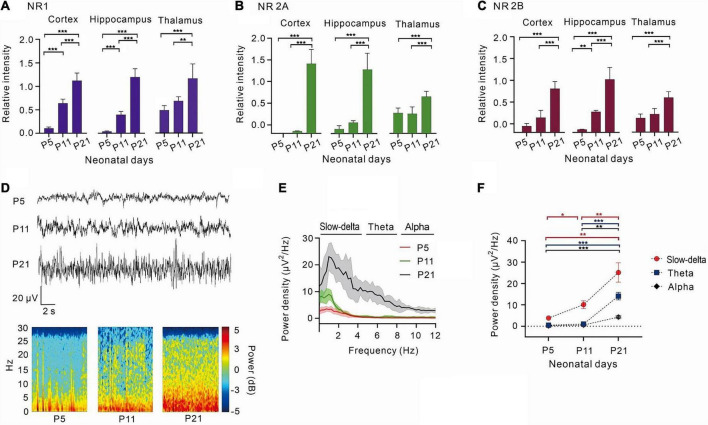
With the development of the central nervous system, NR1, NR2A, NR2B increased gradually, and the power density of theta oscillations gradually strengthens. **(A)** The relative expression of NR1 in the cortex (*n* = 5), hippocampus (*n* = 5), and thalamus (*n* = 5). **(B)** The relative expression of 2A in the cortex (*n* = 5), hippocampus (*n* = 5), and thalamus (*n* = 5). **(C)** The relative intensity of 2B in the cortex (*n* = 5), hippocampus (*n* = 5), and thalamus (*n* = 5). **(D)** EEG and it’s power spectrogram of P5, P11, P21 rats in resting state. **(E)** Power spectral density of cortical EEG at each age (*n* = 5). **(F)** Power density of EEG in different frequency bands from P5 to P21 (*n* = 5). Data are presented as mean ± SD. *P < 0.05; **P < 0.01; ***P < 0.001 by ordinary one-way ANOVA **(A–C)**, or two-way ANOVA **(F)**.

With the development of the central nervous system, the expression of the NMDA receptor subunits, NR1, NR2A, and NR2B, gradually increased (Figures 1A-C). NR1, the most important subunit of NMDA receptors, gradually increased in the cortex (*P* < 0.001, one-way ANOVA, *n* = 5),hippocampus (*P* < 0.001, one-way ANOVA, *n* = 5), and thalamus (*P* < 0.001, one-way ANOVA, *n* = 5) (Figure 1A). NR2A and NR2B levels were low in the cortex and hippocampus of P5 and P11 rats and showed a significant increase in P21 rats (NR2A: < 0.001, one-way ANOVA, *n* = 5; 2B: < 0.001 one-way ANOVA, *n* = 5). However, the growth of NR2A and NR2B was relatively slow in the thalamus (NR2A: *P* < 0.001, one-way ANOVA, *n* = 5; NR2B: *P* < 0.001, one-way ANOVA, *n* = 5) (Figures 1B, C).

By measuring the cortical EEGs of P5, P11, and P21 rats, it was observed that the power density of EEG gradually increased with age, and slow-delta, theta, and alpha oscillations gradually emerged with age (Figures 1D–F). Between days 5 and 11 after birth, slow-delta oscillations appeared in rats, but theta and alpha oscillations were almost absent (slow-delta: *P* = 0.014, two-way ANOVA, *n* = 5; theta: *P* = 0.068, two-way ANOVA, *n* = 5; alpha: *P* = 0.122, two-way ANOVA, *n* = 5). However, both slow-delta and theta oscillations of cortical EEG were significantly observed from day 11 to day 21 in rats, but the change in alpha oscillations of P11-P21 rats was still small (slow-delta: *P* = 0.006, two-way ANOVA, *n* = 5; theta: *P* < 0.001, two-way ANOVA, *n* = 5; alpha: *P* = 0.002, two-way ANOVA, *n* = 5) (Figure 1F).

Knockdown of NR1 enhanced the sedative potency of volatile anesthetics but had no effect on immobility.

**FIGURE 2:**
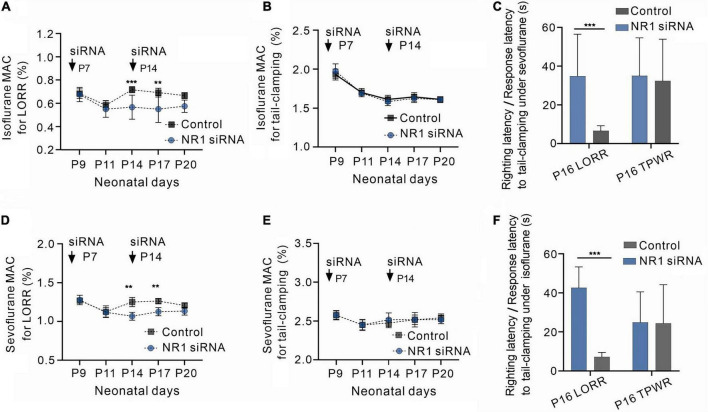
Increased sensitivity to volatile anesthetics in NR1 knockdown rats for the loss of righting reflex and unchanged sensitivity of NR1 knockdown rats for anesthetic-induced immobilization. **(A)** Effects of NR1-siRNA on MAC_LORR_ of isoflurane in rats at various postnatal ages (*n* = 6). **(B)** Effects of NR1-siRNA on MAC_immobility_ of isoflurane in rats at different postnatal ages (*n* = 6). **(C)** Time for the recovery from loss of righting reflex (LORR) and the movement to tail-clamp reflex under isoflurane in P16 rats (left panel, 0.4% isoflurane, *n* = 6; right panel, 1.4% isoflurane, *n* = 6). **(D)** Effects of NR1-siRNA on MAC_LORR_ of sevoflurane in rats at distinct postnatal ages (*n* = 6). **(E)** Effects of NR1-siRNA on MAC_immobility_ of sevoflurane in rats at different postnatal ages (*n* = 6). **(C)** Time for the recovery from loss of righting reflex (LORR) and the movement to tail-clamp reflex under sevoflurane in P16 rats (left panel, 1.0% sevoflurane, *n* = 6; right panel, 2.3% sevoflurane, *n* = 6). Data are presented as mean ± SD. **P < 0.01; ***P < 0.001 by two-way ANOVA **(A,B,D,E)** and unpaired *t*-test **(C,F)**.

Volatile anesthesia was performed after the knockdown of NR1 by intraventricular injection of NR1-siRNA in rats. The 488-positive fluorescence carried by si-RNA was widely present in forebrain (Supplementary Figure 1). It was observed that P14 and P17 rats with isoflurane anesthesic after knockdown of NR1 had significantly lower MAC_LORR_ than the control group (P14: NR1-siRNA group:0.57% ± 0.1%; control group:0.70% ± 0.03%, *P* = 0.003; P17: NR1-siRNA group:0.55% ± 0.11%; control group:0.68% ± 0.04%, *P* = 0.005, two-way ANOVA followed by Sidak’s multiple comparisons test, *n* = 6, Figure 2A). Under sevoflurane, the MAC_LORR_ of P14 and P17 rats in the NR1-siRNA group was also lower than that of the control group (P14: NR1-siRNA group:1.07% ± 0.05%; control group:1.26% ± 0.07%, P = 0.002; P17: NR1-siRNA group:1.13% ± 0.05%; control group:1.26% ± 0.04%, P = 0.003, two-way ANOVA followed by Sidak’s multiple comparisons test, *n* = 6, Figure 2D).

The MAC_LORR_ of the NR1-siRNA group before P11 was not different from that of the control group (P9: NR1-siRNA group:0.68% ± 0.06%; control group:0.69% ± 0.04%, *P* = 0.996; P11: NR1-siRNA group:0.55% ± 0.07%; control group:0.58% ± 0.05%, *P* = 0.974, two-way ANOVA followed by Sidak’s multiple comparisons test, *n* = 6, Figure 2A) under isoflurane, probably due to the low expression of NR1 in the central nervous system. Under sevoflurane, there was also no difference in MAC_LORR_ of P9 and P11 between the NR1-siRNA and control groups (P9: NR1-siRNA group:1.28% ± 0.06%; control group:1.28% ± 0.04%, *P* > 0.999; P11: NR1-siRNA group:1.12% ± 0.06%; control group:1.14% ± 0.08%, *P* = 0.982, two-way ANOVA followed by Sidak’s multiple comparisons test, *n* = 6, Figure 2D). For P20 rats, there was no statistical difference in MAC_LORR_ under isoflurane between the NR1-siRNA group (P20:0.58% ± 0.05%) and the control group (P20:0.66% ± 0.04%, *P* = 0.159 two-way ANOVA followed by Sidak’s multiple comparisons test, *n* = 6, Figure 2A). In the experiment on withdrawal response to tail clamping, there was no difference in MAC_immobility_ between the NR1-siRNA group and control groups of all ages (isoflurane: *P* = 0.726, two-way ANOVA, *n* = 6; sevoflurane: *P* = 0.776, two-way ANOVA, *n* = 6, Figures 2B, E).

Since NR1-siRNA had the most obvious effect between P14 and P17, we selected P16 rats to measure their righting reflex latency and response latency to tail clamping. As expected, NR1-siRNA increased the righting latency under 0.4% isoflurane (34.8 ± 16.5 vs. 6.7 ± 3.5 s, *P* = 0.002, unpaired *t*-test, *n* = 6, Figure 2C) and 0.9% sevoflurane (42.6 ± 12.2 s vs. 7.2 ± 5.9 s, *P* < 0.001, unpaired *t*-test, *n* = 6, Figure 2F) in P16 rats but had no effect on response latency to tail-clamping under 1.4% isoflurane (35.2 ± 11.5 vs. 32.3 ± 8.1 s, *P* = 0.633, unpaired *t*-test, *n* = 6, Figure 2C) and 2.3% sevoflurane (24.8 ± 8.0s vs. 24.3 ± 13.6 s, *P* = 0.940, unpaired *t*-test, *n* = 6, Figure 2F).

Changes in EEG oscillations in P5 and P21 rats treated with different concentrations of volatile anesthetics.

**FIGURE 3:**
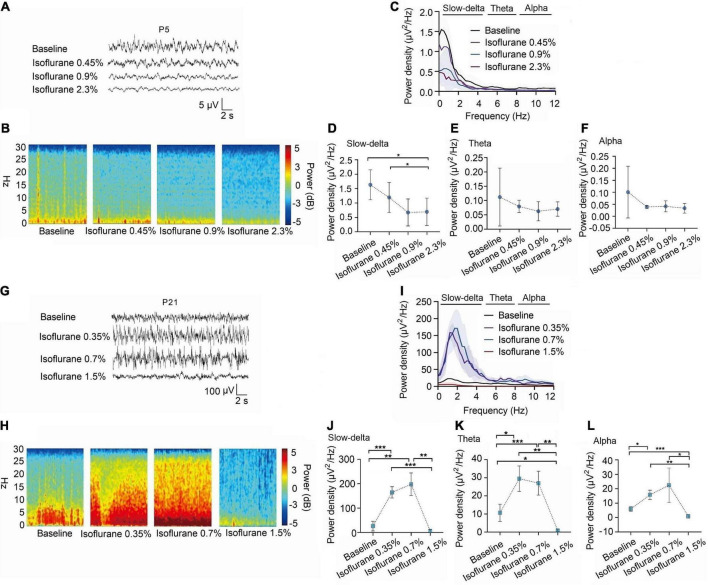
In P5 rats, isoflurane reduced the power density of slow-delta oscillations, while in P21 rats, it increased the power density of slow-delta, theta and alpha oscillations. **(A,B)** EEG and it’s power spectrogram of P5 rats under 50%MAC_LORR_, MAC_LORR_ and MAC_immobility_ of isoflurane. **(G,H)** EEG and it’s power spectrogram of P21 rats under 50%MAC_LORR_, MAC_LORR_ and MAC_immobility_ of isoflurane. **(C,I)** EEG power spectral density of P5, P21 rats at elevated isoflurane concentrations (*n* = 5). **(D–F)** The change of power density of slow-delta, theta and alpha oscillations on P5 rats. **(J–L)** The change of power density of slow-delta, theta and alpha oscillations on P21 rats. Data are presented as mean ± SD. *P < 0.05, **P < 0.01, ***P < 0.001 by repeated measures one-way ANOVA **(D–F, J–L)**.

We observed EEG in p5 and p21 rats at different concentrations of inhalation anesthetics (Figures 3A–C,G–I). Under isoflurane, slow-delta oscillations in P5 rats were inhibited (baseline:1.63 ± 0.52 μV^2^ Hz^–1^, 0.45% isoflurane:1.19 ± 0.52 μV^2^ Hz^–1^, 0.9% isoflurane:0.67 ± 0.47 μV^2^ Hz^–1^, 2.3% isoflurane:0.55 ± 0.47 μV^2^ Hz^–1^, P = 0.029 by repeated measures one-way ANOVA, *n* = 5, Figure 3D), however, changes of theta oscillations (baseline:0.11 ± 0.10 μV^2^ Hz^–1^, 0.45% isoflurane:0.08 ± 0.02 μV^2^ Hz^–1^, 0.9% isoflurane:0.06 ± 0.03 μV^2^ Hz^–1^, 2.3% isoflurane:0.07 ± 0.03 μV^2^ Hz^–1^, P = 0.429 by repeated measures one-way ANOVA, n = 5, Figure 3E) and alpha oscillations (baseline:0.10 ± 0.11 μV^2^ Hz^–1^, 0.45% isoflurane:0.04 ± 0.01 μV^2^ Hz^–1^, 0.9% isoflurane:0.04 ± 0.22 μV^2^ Hz^–1^, 2.3% isoflurane:0.03 ± 0.20 μV^2^ Hz^–1^, *P* = 0.283 by repeated measures one-way ANOVA, *n* = 5, Figure 3F) in P5 rats were not significant. For P21 rats, the power density of slow-delta (baseline:26.48 ± 19.90 μV^2^ Hz^–1^, 0.35% isoflurane:164.44 ± 23.05 μV^2^ Hz^–1^, 0.7% isoflurane:197.78 ± 46.30 μV^2^ Hz^–1^, 1.5% isoflurane:6.34 ± 5.88 μV^2^ Hz^–1^, *P* < 0.001 by repeated measures one-way ANOVA, *n* = 5, Figure 3J), theta (baseline:10.65 ± 4.74 μV^2^ Hz^–1^, 0.35% isoflurane:29.49 ± 7.05 μV^2^ Hz^–1^, 0.7% isoflurane:27.01 ± 6.58 μV^2^ Hz^–1^, 1.5% isoflurane:0.86 ± 0.58 μV^2^ Hz^–1^, *P* < 0.001 by repeated measures one-way ANOVA, *n* = 5, Figure 3K), and alpha (baseline:5.92 ± 1.54 μV^2^ Hz^–1^, 0.35% isoflurane:15.75 ± 3.24 μV^2^ Hz^–1^, 0.7% isoflurane:22.42 ± 11.91 μV^2^ Hz^–1^, 1.5% isoflurane:0.88 ± 1.24 μV^2^ Hz^–1^, *P* = 0.022 by repeated measures one-way ANOVA, *n* = 5, Figure 3L) oscillations was enhanced and then suppressed with increasing isoflurane concentration.

**FIGURE 4:**
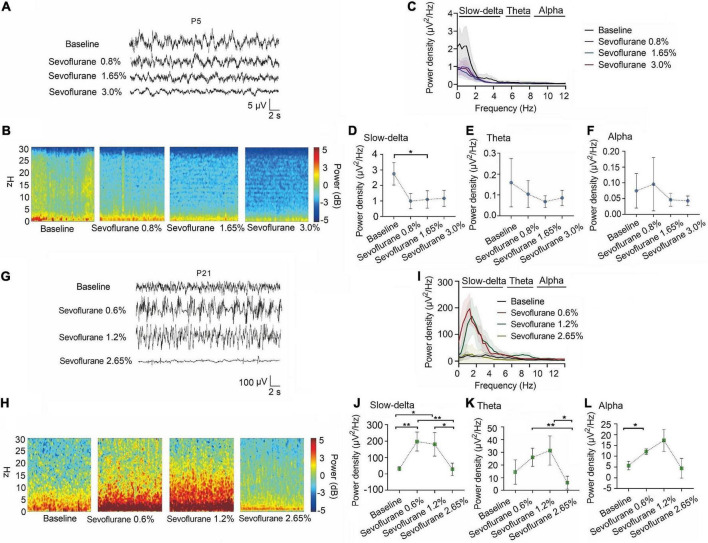
Slow-delta oscillations was suppressed in P5 rats with increasing sevoflurane concentrations, while enhanced slow-delta, theta and alpha oscillations in P21 rats. **(A,B)** EEG and it’s power spectrogram of P5 rats under 50%MAC_LORR_, MAC_LORR_ and MAC_immobility_ of sevoflurane. **(G,H)** EEG and it’s power spectrogram of P21 rats under 50%MAC_LORR_, MAC_LORR_ and MAC_immobility_ of sevoflurane. **(C,I)** EEG power spectral density of P5, P21 rats under increased concentrations of sevoflurane concentrations (*n* = 5). **(D–F)** The change of power density of slow-delta, theta and alpha oscillations on P5 rats. **(J–L)** The change of power density of slow-delta, theta and alpha oscillations on P21 rats. Data are presented as mean ± SD. *P < 0.05, **P < 0.01 by repeated measures one-way ANOVA **(D–F, J–L)**.

A similar phenomenon was observed with sevoflurane. The power density of slow-delta oscillations in P5 rats was suppressed with increasing sevoflurane concentration (baseline:2.75 ± 0.73 μV^2^ Hz^–1^, 0.8% sevoflurane:1.13 ± 0.46 μV^2^ Hz^–1^, 1.65% sevoflurane:0.96 ± 0.57 μV^2^ Hz^–1^, 3.0% sevoflurane:1.17 ± 0.52 μV^2^ Hz^–1^, P = 0.015 by repeated measures one-way ANOVA, *n* = 5, Figure 4D). The power density of theta (baseline:0.16 ± 0.12 μV^2^ Hz^–1^, 0.8% sevoflurane:0.10 ± 0.06 μV^2^ Hz^–1^, 1.65% sevoflurane:0.07 ± 0.03 μV^2^ Hz^–1^, 3.0% sevoflurane:0.09 ± 0.04 μV^2^ Hz^–1^, *P* = 0.285 by repeated measures one-way ANOVA, *n* = 5, Figure 4E) and alpha oscillations (baseline:0.07 ± 0.05 μV^2^ Hz^–1^, 0.8% sevoflurane:0.10 ± 0.08 μV^2^ Hz^–1^, 1.65% sevoflurane:0.05 ± 0.02 μV^2^ Hz^–1^, 3.0% sevoflurane:0.04 ± 0.02 μV^2^ Hz^–1^, P = 0.356 by repeated measures one-way ANOVA, *n* = 5, Figure 4F) in P5 rats did not change significantly. For P21 rats, the power density of slow-delta oscillations increased under 0.6% and 1.2% sevoflurane but decreased under 2.65% sevoflurane (baseline:30.97 ± 12.51 μV^2^ Hz^–1^, 0.6% sevoflurane:197.34 ± 57.95 xμV^2^ Hz^–1^, 1.2% sevoflurane:180.59 ± 71.98 μV^2^ Hz^–1^, 2.65% sevoflurane:27.94 ± 17.63 μV^2^ Hz^–1^, *P* < 0.001 by repeated measures one-way ANOVA, *n* = 5, Figure 4J). Under sevoflurane, the power density of theta oscillations in P21 rats was enhanced until a high concentration (baseline:14.42 ± 9.65 μV^2^ Hz^–1^, 0.6% sevoflurane:26.08 ± 7.19 μV^2^ Hz^–1^, 1.2% sevoflurane:31.49 ± 7.19μV^2^ Hz^–1^, 2.65% sevoflurane:6.05 ± 5.09 μV^2^ Hz^–1^, *P* = 0.007 by repeated measures one-way ANOVA, *n* = 5, Figure 4K). The power density of alpha oscillations was low, but with the increase of sevoflurane concentration, the alpha oscillations still increased first and then decreased (baseline:5.70 ± 1.98 μV^2^ Hz^–1^, 0.6% sevoflurane:12.11 ± 1.30 μV^2^ Hz^–1^, 1.2% sevoflurane:17.35 ± 6.40 μV^2^ Hz^–1^, 2.65% sevoflurane:4.40 ± 8.95 μV^2^ Hz^–1^, *P* = 0.005 by repeated measures one-way ANOVA, *n* = 5, Figure 4L). We further found that the change trends in slow-delta, theta, and alpha oscillations in female and male rats of all ages were consistent under inhalation anesthesia, although several indicators were not statistically significant due to the small sample size (Supplementary Figures 2, 3).

Volatile anesthetics suppressed the power density of EEG in P21 rats after knockdown of NR1.

**FIGURE 5:**
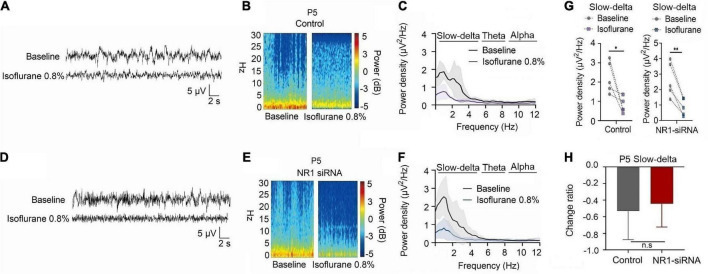
Knockdown of NR1 did not change the effect of slow-delta oscillations in P5 rats under isoflurane anesthesia. **(A,B,D,E)** EEG and it’s power spectrogram of P5 rats under isoflurane in the control group and NR1-siRNA group. **(C,F)** EEG power spectral density of P5 rats under isoflurane in the control group and NR1-siRNA group (*n* = 5). **(G)** The change of power density of slow-delta oscillations of P5 rats under isoflurane in the control group and NR1-siRNA group (*n* = 5). **(H)** The change ratio of the power density of slow-delta, theta and alpha oscillations of P5 rats under isoflurane (*n* = 5). Data are presented as mean ± SD. n.s., not significant; *P < 0.05, **P < 0.01 by paired *t*-test **(G)** and unpaired *t*-test **(H)**.

**FIGURE 6:**
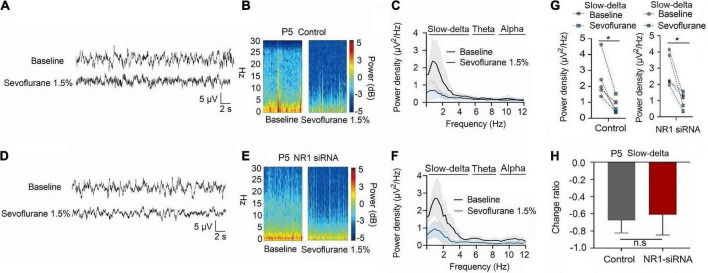
Knockdown of NR1 did not change the effect of slow-delta oscillations in P5 rats under sevoflurane anesthesia. **(A,B,D,E)** EEG and it’s power spectrogram of P5 rats under sevoflurane in the control group and NR1-siRNA group. **(C,F)** EEG power spectral density of P5 rats under sevoflurane in the control group and NR1-siRNA group (*n* = 5). EEG power spectral density. **(G)** The change of power density of slow-delta oscillations of P5 rats under sevoflurane in the control group and NR1-siRNA group (*n* = 5). **(H)** The change ratio of the power density of slow-delta oscillations of P5 rats under sevoflurane (*n* = 5). Data are presented as mean ± SD. n.s., not significant; *P < 0.05 by paired *t*-test **(G)** and unpaired *t*-test **(H)**.

For P5 rats, because the expression of NR1 is very low, NR1-siRNA had very little effect on NR1 content in the cortex, hippocampus, and thalamus (cortex: *P* = 0.457; hippocampus: *P* = 0.846; thalamus: *P* = 0.215 by unpaired *t*-test, *n* = 5, Supplementary Figures 4A–C). Under isoflurane, the power density of slow-delta of P5 rats decreased in both the control group (slow-delta: *P* = 0.040, paired *t*-test, *n* = 5, Figure 5G) and the NR1-siRNA group (slow-delta: P = 0.004, paired *t*-test, *n* = 5, Figure 5G). In P5 rats, the changed ratio of slow-delta oscillations in the control group was −0.58 ± 0.32, while the changed ratio of slow-delta oscillations was −0.68 ± 0.12 in the NR1-siRNA group (slow-delta: *P* = 0.523, unpaired *t*-test, *n* = 5, Figure 5H). Under sevoflurane, the power density of slow-delta oscillations of P5 rats also decreased in both the control group (slow-delta: *P* = 0.017, paired *t*-test, *n* = 5, Figure 6G) and the NR1-siRNA group (slow-delta: *P* = 0.020, paired *t*-test, *n* = 5, Figure 6G). Under sevoflurane, the changed ratio of slow-delta oscillations in the control group of P5 rats was −0.68 ± 0.15, and the changed ratio of slow-delta oscillations was −0.61 ± 0.24 in the NR1-siRNA group (slow-delta: *P* = 0.624, unpaired *t*-test, *n* = 5, Figure 6H).

**FIGURE 7:**
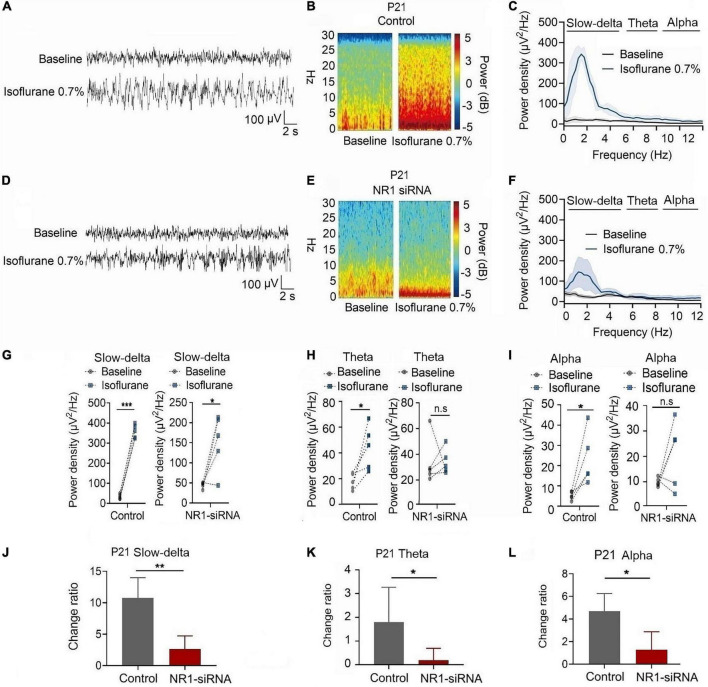
Knockdown of NR1 inhibited the enhancement of slow-delta, theta and alpha oscillations in P21 rats. **(A,B,D,E)** EEG and it’s power spectrogram of P21 rats under isoflurane in the control group and NR1-siRNA group. **(C,F)** EEG power spectral density of P21 rats under isoflurane in the control group and NR1-siRNA group (*n* = 5). **(G–I)** The change of power density of slow-delta, theta and alpha oscillations of P21 rats under isoflurane in the control group and NR1-siRNA group (*n* = 5). **(J–L)** The change ratio of the power density of slow-delta, theta and alpha oscillations of P21 rats under isoflurane (*n* = 5). Data are presented as mean ± SD. n.s., not significant; *P < 0.05, **P < 0.01, ***P < 0.001 by paired *t*-test **(G–I)** and unpaired *t*-test **(J–L)**.

**FIGURE 8:**
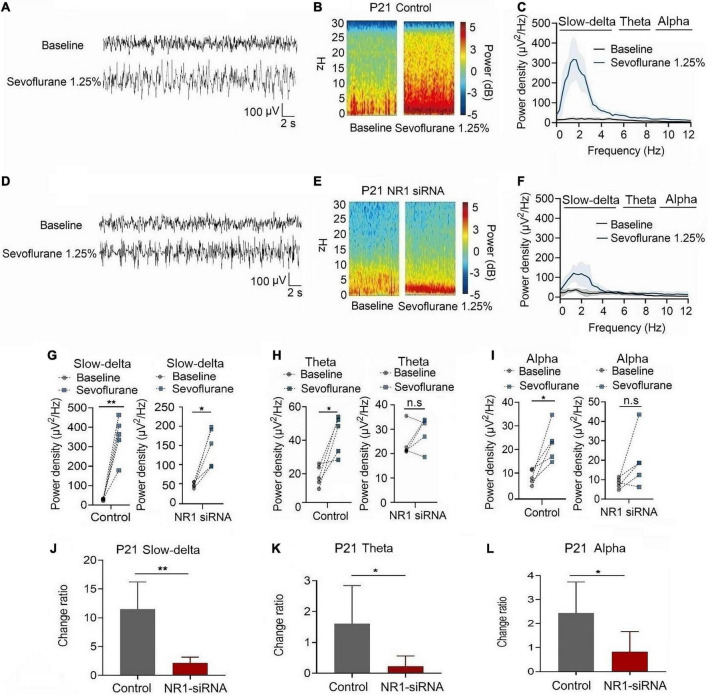
Knockdown of NR1 inhibited the enhancement of slow-delta, theta and alpha oscillations in P21 rats. **(A,B,D,E)** EEG and it’s power spectrogram of P21 rats under sevoflurane in the control group and NR1-siRNA group. **(C,F)** EEG power spectral density of P21 rats under sevoflurane in the control group and NR1-siRNA group (*n* = 5). EEG power spectral density. **(G–I)** The change of power density of slow-delta, theta and alpha oscillations of P21 rats under sevoflurane in the control group and NR1-siRNA group (*n* = 5). **(J–L)** The change ratio of the power density of slow-delta, theta and alpha oscillations of P21 rats under sevoflurane (*n* = 5). Data are presented as mean ± SD. n.s., not significant; *P < 0.05, **P < 0.01 by paired *t*-test **(G–I)** and unpaired *t*-test **(J–L)**.

In P21 rats, the expression of NR1 in the cortex, hippocampus, and thalamus was significantly reduced by the injection of NR1-siRNA into the ventricle (cortex: *P* = 0.004; hippocampus: *P* < 0.001; thalamus: *P* = 0.028 by unpaired *t*-test, *n* = 5, Supplementary Figures 1D–F). Through EEG recordings, we observed that isoflurane enhanced the power density of slow-delta, theta, and alpha oscillations in the control group (slow-delta: *P* < 0.001; theta: *P* = 0.036; alpha: *P* = 0.032 by paired *t*-test, *n* = 5, Figures 7G–I). In the NR1-siRNA group, isoflurane also increased the power density of slow-delta oscillations (*P* = 0.032 by paired *t*-test, *n* = 5, Figure 7G) but had no effect on theta and alpha oscillations (theta: *P* = 0.952; alpha: *P* = 0.164 by paired *t*-test, *n* = 5, Figures 7H, I). Under sevoflurane, the effect of the power density of slow-delta, theta, and alpha oscillations was similar to that elicited by isoflurane. The power density of slow-delta, theta, and alpha oscillations was enhanced in the control group (slow-delta: *P* = 0.003; theta: *P* = 0.020; alpha: *P* = 0.021 by paired *t*-test, *n* = 5, Figures 8G–I). In the NR1-siRNA group, sevoflurane also increased the power density of slow-delta oscillations (*P* = 0.010 by paired *t*-test, *n* = 5, Figure 8G), but had no effect on theta and alpha oscillations (theta: *P* = 0.224; alpha: *P* = 0.123 by paired *t*-test, *n* = 5, Figures 8H, I).

Under isoflurane, the changed ratios of slow-delta, theta, and alpha oscillations in the control group of P21 rats were 10.78 ± 3.22, 1.81 ± 1.46, and 4.08 ± 1.93, respectively, and the changed ratios of slow-delta, theta, and alpha oscillations were 2.65 ± 2.08, 0.20 ± 0.50, and 1.29 ± 1.58, respectively, in the NR1-siRNA group (slow-delta: *P* = 0.001; theta: *P* = 0.048; alpha: *P* = 0.037 by unpaired *t*-test, *n* = 5, Figures 7J–L). Under sevoflurane, the changed ratios of slow-delta, theta, and alpha oscillations in the control group of P21 rats were 11.50 ± 4.72, 1.61 ± 1.24, and 2.44 ± 1.30, respectively; and the changed ratios of slow-delta, theta, and alpha oscillations were 2.14 ± 1.05, 0.23 ± 0.33, and 0.83 ± 0.84, respectively, in the NR1-siRNA group (slow-delta: *P* = 0.003; theta: *P* = 0.043; alpha: *P* = 0.048 by unpaired *t*-test, *n* = 5, Figures 8J–L).

## Discussion

Our study showed that the NR1, NR2A, and NR2B subunits of NMDA receptors gradually increased in the cortex, hippocampus, and thalamus with increasing age and were accompanied by a gradual enhancement of slow-delta, theta, and alpha oscillations. In rats before P14, knockdown of NR1 did not affect the sedative effect of volatile anesthetics. Inhalation anesthesia also did not increase the power density of the slow-delta, theta, and alpha oscillations in P5 rats. In rats 14 days after birth, knockdown of NR1 enhanced the hypnotic actions of volatile anesthetics. Similarly, volatile anesthetics suppressed the power density of slow-delta, theta, and alpha oscillations in P21 rats following NR1 knockdown. It is worth noting that our behavioral experiments showed that there was no difference between the NR1-siRNA and control groups in the withdrawal response to the tail clamping experiment that explored the effect on immobility. These results suggest that the sedative effect of inhalation anesthesia requires the involvement of NMDA receptors, and the NMDA receptors play an important role in enhancing theta oscillations induced by inhalation anesthesia.

In immature rats, MAC_LORR_ changes with age. Therefore, we used various concentrations of volatile anesthetics in different age groups. In the EEG experiment, the concentrations of volatile anesthetics in P5 and P21 rats were selected according to the MAC_LORR_ measured in the LORR experiment. In the behavioral experiment, the MAC_LORR_ of P9 to P11 gradually decreased, and the expression of NMDA receptors was low during this period, which may be due to other mechanisms. We hypothesized that this may be related to the development of the GABAergic system. Previous studies have shown that GABA development undergoes an excitation-inhibitory functional transition (Ben-Ari, 2002). GABA exhibits polarized excitability at an early stage of development, which increases neuronal excitability. During development, GABA gradually shows a hyperpolarized inhibitory effect, which decreases neuronal excitability (Kirmse et al., 2015). Maturation of the GABAergic system occurs approximately 2 weeks after birth in rats (Dzhala et al., 2005). Therefore, the MAC_LORR_ of the P9 rats was higher than that of the P11 rats. Subsequently, we found that the MAC_LORR_ of rats after P11 gradually increased, which may be due to the increased excitability of neurons caused by the development of NMDA receptors. However, MAC_immobility_ did not increase significantly in the rats after P11. It is possible that the development of NMDA receptors may not affect MAC_immobility_ of inhalation anesthesia, which can also be verified by ventricle injection of NR1-siRNA. Our experiments showed that the circuitry mediating the tail clamp withdrawal motor response is independent of GluR1-containing NMDA receptors. Previous studies have shown that the spinal cord mediates most of the ability of inhaled anesthetics to produce immobility (Antognini and Carstens, 1998, Sonner et al., 2003). However, the mechanism by which inhalation anesthesia affects immobility remains unclear.

There are several main targets for the hypnotic effects of general anesthesia, including the cortex, thalamus, and brainstem (Leung et al., 2014). Hypnotic effects are often associated with brain oscillations recorded by electroencephalography under anesthesia. Notably, the thalamus and hippocampus are closely involved in theta oscillation generation (Malver et al., 2014). Although theta oscillations can also be found in the subicular complex, entorhinal cortex, perirhinal cortex, and cingulate cortex, none of these structures alone generate theta oscillations (Pare and Collins, 2000). However, the enhancement of slow-delta under anesthesia is related to the inhibition of excitatory inputs from the thalamus and brainstem to the cortex (Lewis et al., 2012). Alpha oscillations are generated by thalamocortical feedback loops (Klimesch, 1997). Therefore, in this study, ventricle-injection siRNA was used to extensively intervene in NR1 expression in various parts of the brain to better explore the effect of NMDA development on EEG oscillations under volatile anesthetics. NMDA receptors are one of the main targets of inhaled anesthetic drugs. Therefore, the effect of inhalation anesthesia on theta oscillations in the EEG through NMDA receptors requires further study.

In human, previous study showed that slow-delta is present in the EEG at all ages after birth, but theta and alpha oscillations appear in the 4 months of life (Cornelissen et al., 2015). In our study, slow-delta oscillations appeared first in the EEG of P5 rats, followed by theta and alpha oscillations. Therefore, the emerging sequence of the component frequency in the EEG is similar between humans and rats. Accordingly, the expression pattern of NMDA receptors between humans and rats is also similar. The expression levels of NR1, NR2A, and NR2B increased in the human fetal cerebral cortex during the second trimester of gestation, and NR1 developed earlier than NR2A and NR2B (Bagasrawala et al., 2017). In rat brain, NR1 expression starts as early as E14, reaches a peak at approximately the third week of life, and then decreases lightly to adult levels (Laurie and Seeburg, 1994). Other subtypes broadly follow this pattern of NR1 development (Paupard et al., 1997). Collectively, the similarity in the development of EEG oscillations and NMDA expression between humans and rats suggests a potential clinical translation.

This study had some limitations. First, we focused on rats on the 5th and 21st days of birth, two extreme time points. However, we did not perform experimental observations in rats between P5 and P21. Second, although we verified the expression of NMDA subunits in different parts of the central nervous system, we did not examine the effects of NMDA development on neurons at the electrophysiological level.

In summary, our findings support the concept that NMDA receptors may be necessary for the enhancement of cortical EEG oscillations by volatile anesthetics.

## Data availability statement

The original contributions presented in this study are included in the article/Supplementary material, further inquiries can be directed to the corresponding authors.

## Ethics statement

The animal study was reviewed and approved by Ethics Committee of West China Hospital of Sichuan University.

## Author contributions

MZ, DZ, and TZ contributed to the conception and design of the study. MZ, YC, and YY performed the experiments and statistical analysis. MZ, YC, DZ, and TZ wrote the draft of the manuscript. All authors contributed to the manuscript revision, read, and approved the submitted version.
