# Supplementary material for: Development of NMDA receptors contributes to the enhancement of electroencephalogram oscillations under volatile anesthetics in rats
Source: Front Neural Circuits. 2022 Dec 15;16:1065374. doi: 10.3389/fncir.2022.1065374 (PMC9797678; doi:10.3389/fncir.2022.1065374)
Supplement: Supplementary file 1 [file Data_Sheet_1.docx]

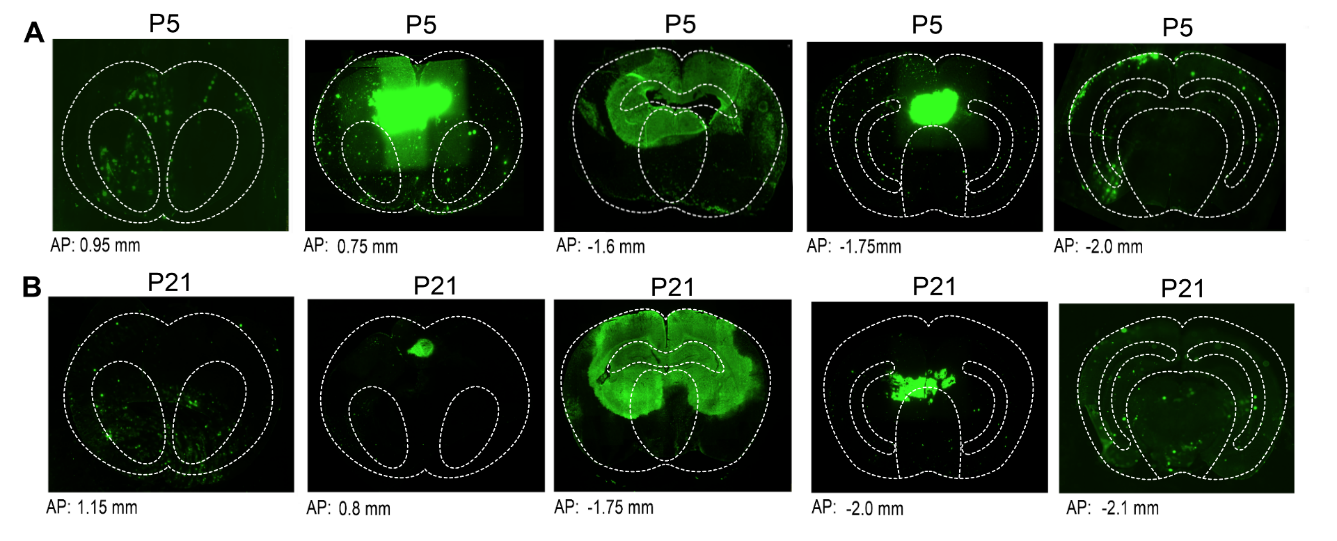


Supplementary Figure 1. Diffusion of NR1-siRNA in the brain after intraventricular injection. (A) Diffusion of NR1-siRNA in P5 rats. (B) Diffusion of NR1-siRNA in P21 rats.


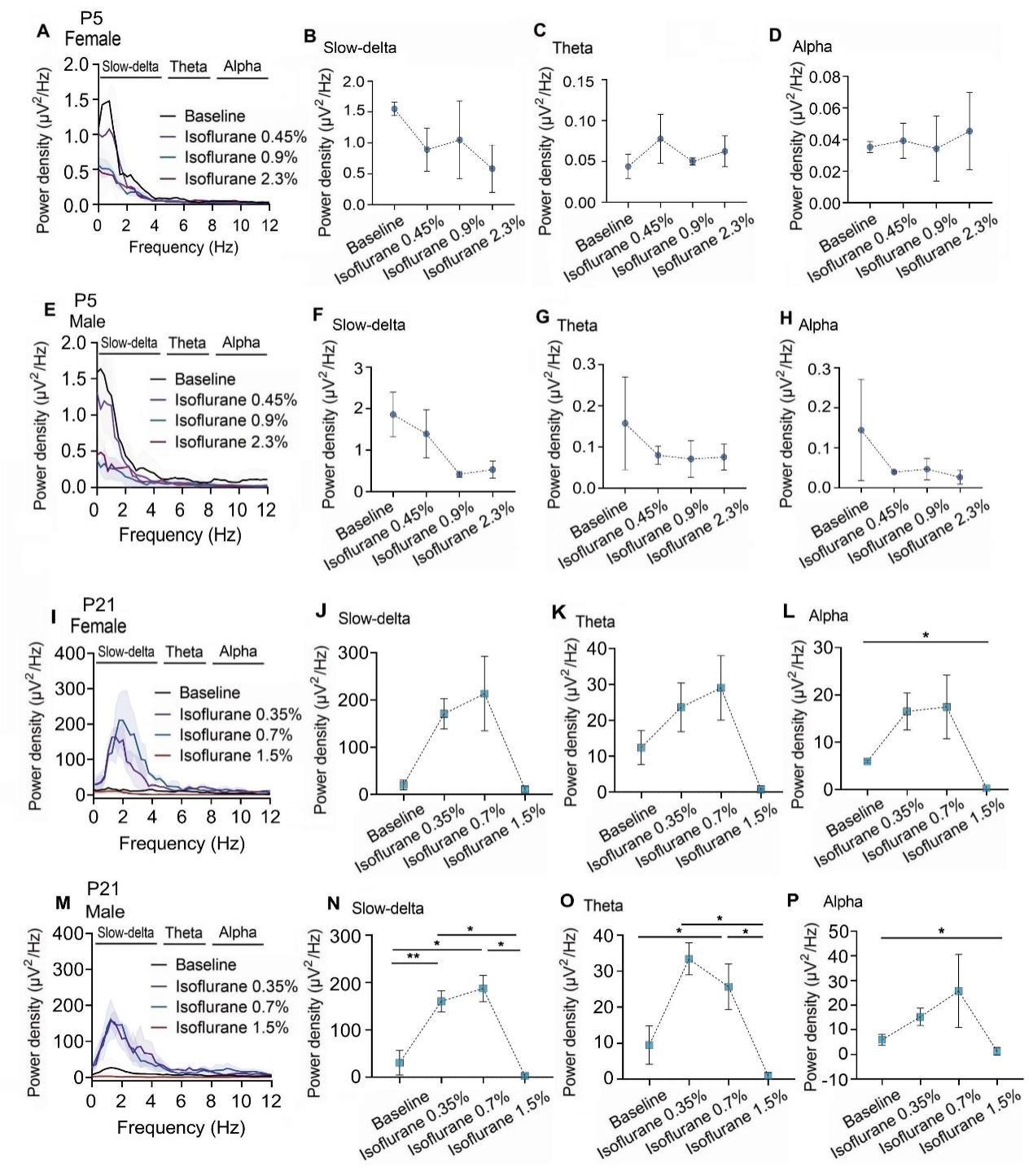


Supplementary Figure 2. Changes in slow-delta, theta and alpha oscillations in female and male rats at P5 and P21 under isoflurane anesthesia. (A, E) EEG power spectral density of female and male rats at P5 under elevated isoflurane concentrations (female n = 2, male n=3). (B, C, D, F, G, H) The changes of power density of slow-delta, theta and alpha oscillations in female and male rats at P5 under 50%MAC_LORR_, MAC_LORR_ and MAC_immobility_ of isoflurane. (I, M) EEG power spectral density of female and male rats at P21 under elevated isoflurane concentrations (female n = 2, male n=3). (J, K, L, N, O, P) The changes of power density of slow-delta, theta and alpha oscillations in female and male rats at P21 under 50%MAC_LORR_, MAC_LORR_ and MAC_immobility_ of isoflurane. Data are presented as mean ± SD. * P<0.05, ** P < 0.01 by repeated measures one-way ANOVA (B, C, D, F, G, H, J, K, L, N, O, P).


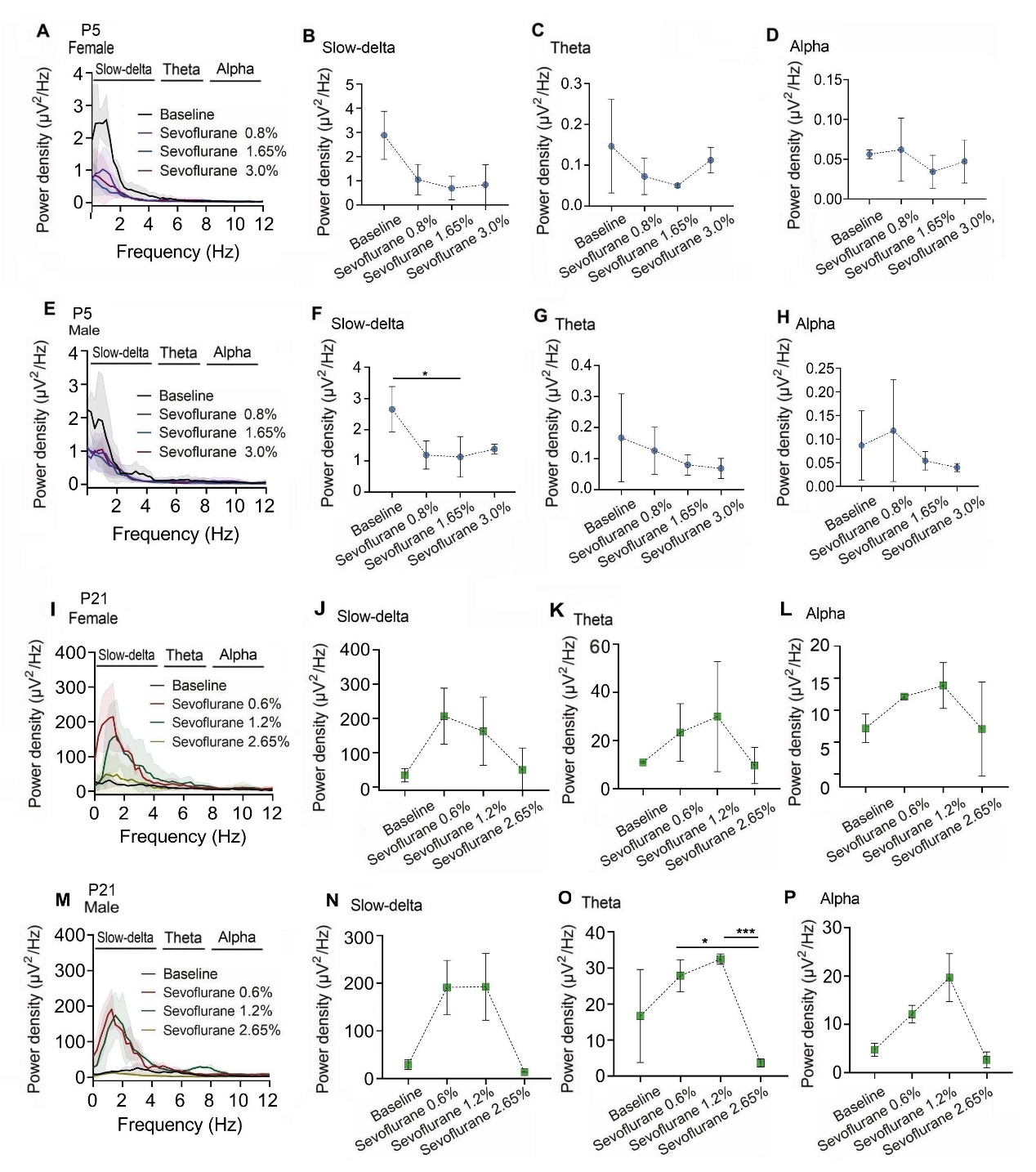


Supplementary Figure 3. Changes in slow-delta, theta and alpha oscillations in female and male rats at P5 and P21 under sevoflurane anesthesia. (A, E) EEG power spectral density of female and male rats at P5 under elevated sevoflurane concentrations (female n = 2, male n=3). (B, C, D, F, G, H) The changes of power density of slow-delta, theta and alpha oscillations in female and male rats at P5 under 50%MAC_LORR_, MAC_LORR_ and MAC_immobility_ of sevoflurane. (I, M) EEG power spectral density of female and male rats at P21 under elevated sevoflurane concentrations (female n = 2, male n=3). (J, K, L, N, O, P) The changes of power density of slow-delta, theta and alpha oscillations in female and male rats at P21 under 50%MAC_LORR_, MAC_LORR_ and MAC_immobility_ of sevoflurane. Data are presented as mean ± SD. * P<0.05, *** P < 0.001 by repeated measures one-way ANOVA (B, C, D, F, G, H, J, K, L, N, O, P).


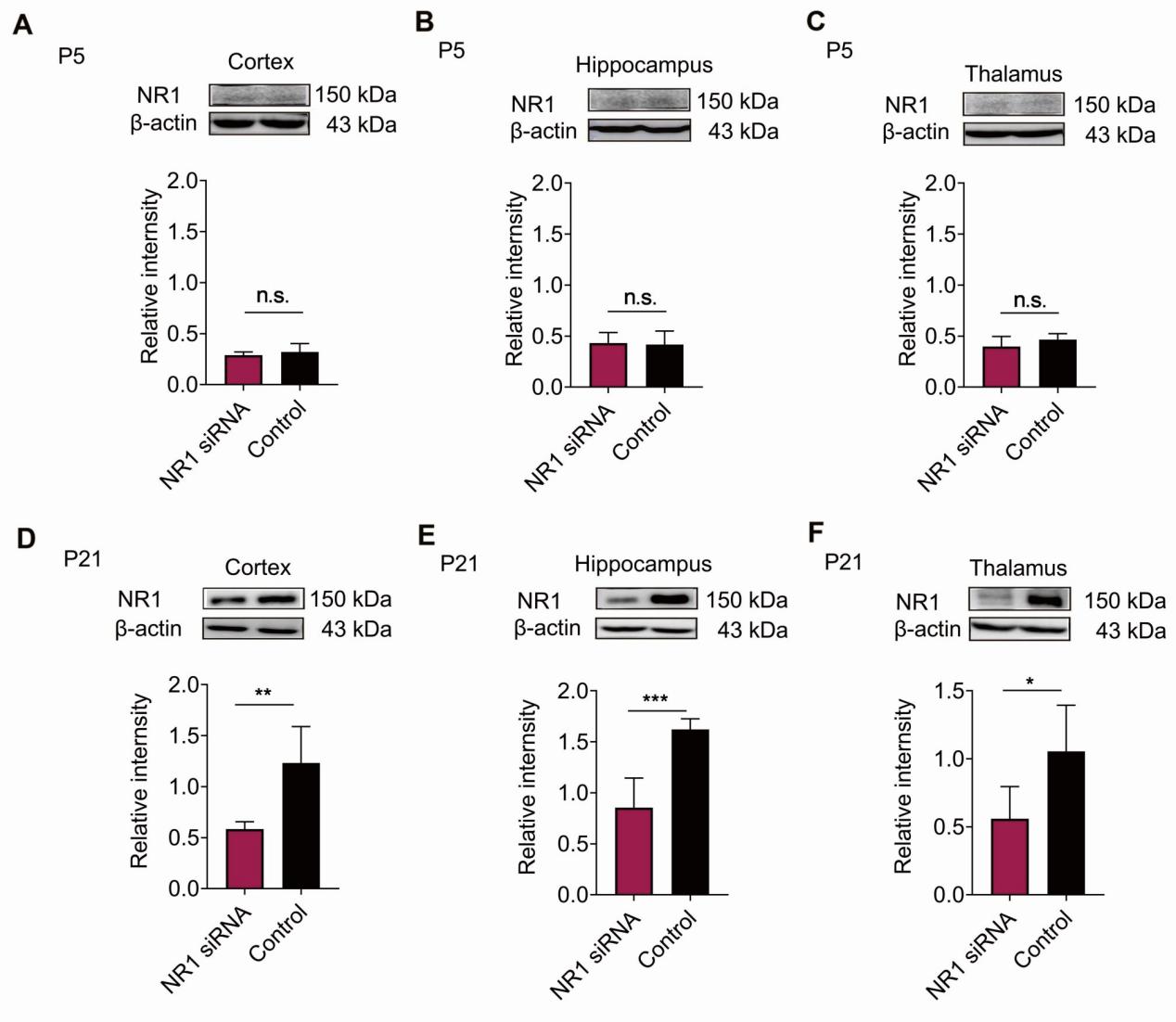


Supplementary Figure 4. Changes of NR1 content in cortex, hippocampus and thalamus after ventricle injection of NR1-siRNA. (A, B, C) For P5 rats, NR1-siRNA do not alter the amount of NR1 in cortex, hippocampus and thalamus (n = 5). (D, E, F) For P21 rats, NR1-siRNA reduce the amount of NR1 in cortex, hippocampus and thalamus (n = 5). Data are presented as mean ± SD. n.s., not significant; * P<0.05, ** P < 0.01, *** P < 0.001 by unpaired t-test.
